# Editorial: Lipid Nanoparticles as a Novel Strategy to Deliver Bioactive Molecules

**DOI:** 10.3389/fchem.2021.655480

**Published:** 2021-03-04

**Authors:** Alan Talevi, Nelson Duran, Guillermo Raul Castro

**Affiliations:** ^1^ Laboratory of Bioactive Research and Development (LIDeB), Department of Biological Sciences, Faculty of Exact Sciences, University of La Plata (UNLP), La Plata, Argentina; ^2^ Consejo Nacional de Investigaciones Científicas y Técnicas (CONICET), Buenos Aires, Argentina; ^3^ Laboratory of Urogenital Carcinogenesis andImmunotherapy, Department of Structural and Functional Biology, Institute of Biology, University of Campinas (UNICAMP), Campinas, Brazil; ^4^ Laboratorio de Nanobiomateriales, Centro de Investigacióny Desarrollo enFermentaciones Industriales (CINDEFI), Departamento de Química, Facultad de Ciencias Exactas, Universidad Nacional deLa Plata (UNLP)-CONICET (CCT La Plata), La Plata, Argentina

**Keywords:** lipid nanoparticles, drug delivery, nanocarriers, lipid nanocarriers, nanotechnology, liposomes, biopharmaceutics, gene delivery

**Editorial on the Research Topic** **Lipid Nanoparticles as a Novel Strategy to Deliver Bioactive Molecules**Lipid nanoparticles are so far among the most successful nanodelivery systems, considering the significant proportion of marketed nanocarriers that correspond to this category. High loading capacity, biocompatibility, and environmentally friendly obtention techniques can be mentioned among their most prominent specific advantages. The current research topic on *Lipid Nanoparticles as a Novel Strategy to Deliver Bioactive Molecules* encompasses original and review articles on a wide range of lipid nanoparticle-related topics, from encapsulation of gene therapies to novel characterization approaches. The collection of articles also expresses the increasing versatility of these nanosystems, owing to the use of hybrid and functionalized carriers.

The review article “Solid lipid nanoparticles for drug delivery: pharmacological and biopharmaceutical aspects” provides a literature survey on original publications from the last 7 years, considering only those articles where pharmacodynamic and/or pharmacokinetic studies have been performed. The article focuses on biopharmaceutical aspects of nanosystems administered through different routes, from oral absorption to enhanced brain penetration, including a critical view on current uncertainties and future directions.

The article “Surface Plasmon Resonance as a Characterization Tool for Lipid Nanoparticles used in Drug Delivery” describes recent work on the use of surface plasmon resonance to predict the potential interactions of lipid nanoparticles with biological proteins, including constituents of the protein corona that may trigger elimination by the mononuclear phagocyte system, thus conditioning their bioavailability.

For their part, Muraca et al. reviewed the use of lipid nanosystems for the delivery of therapeutics against a group of three trypanosomatid-caused neglected diseases: African trypanosomiasis, Chagas disease, and leishmaniasis. The article “Trypanosomatid-caused conditions: State of the art of therapeutics and potential applications of lipid-based nanocarriers” concludes that lipid nanocarriers could improve the efficacy–safety balance of known treatments, diminishing cytotoxicity and organ toxicity, especially in the case of leishmaniasis. However, it also underlines that last generation systems are still to be widely explored in the field of neglected conditions.

In the work entitled “Functional hybrid nanoemulsions for sumatriptan intranasal delivery,” Ribeiro et al. developed nanostructures composed of biopolymers and copaiba essential oil for the delivery of sumatriptan for intranasal administration. The work tried to solve bioavailability issues of that antimigraine active pharmaceutical ingredient. From the biopolymers screened, alginate was selected to produce a nanoemulsion of sumatriptan with the oil. The nanoemulsions displayed high entrapment efficiency in the range of 69 to 41%, high stability during at least for one year, and improved kinetic release compared to free drug. Also, tests on zebrafish larvae did not show changes in behavior either in their morphology.

An interesting work entitled “Optimizing the intracellular delivery of therapeutic anti-inflammatory TNF-α siRNA to activated macrophages using lipidoid-polymer hybrid nanoparticles” presented by Lokras et al. applied quality by design methodology to optimize RNA interference as strategy to silence the overexpression of pro-inflammatory cytokines such as tumor necrosis factor-alpha (TNF-α) induced by macrophages during lung pathologies like chronic obstructive pulmonary disease (COPD). A hybrid platform used for the intracellular delivery of TNF-α siRNA was the lipid-like transfection agent lipidoid L5 and poly(D,L-lactide-co-glycolide) successfully assayed in lipopolysaccharide-activated murine macrophage cell line RAW 264.7. Importantly, kinetic release of fluorescent siRNA from LPNs *in vitro* and *in vivo* demonstrated not only sustained release of siRNA from the nanoparticles but also a correlation between cell uptake and *in vivo* distribution. In a related review (“Advances in Lipid Nanoparticles for mRNA-Based Cancer Immunotherapy”), Guevara et al. summarize recent advances in the development of lipid particles for the delivery of mRNA-based immunotherapies, with a focus on cancer treatment. They also highlight a diversity of immunotherapeutic approaches through mRNA delivery and discuss the main factors affecting *in vivo* transfection efficiency and tropism of mRNA-loaded lipid nanoparticles.

In another relevant work called “Rapamycin-loaded lipid nanocapsules induce selective inhibition of the mTORC1-signaling pathway in glioblastoma cells,” Garcion et al. developed lipid nanocapsules containing rapamycin, a poorly water-soluble inhibitor of mTOR phosphorylation at Ser(2448), for potential application in cancer, particularly in glioblastomas combined with radiotherapy. Inhibition of mTOR phosphorylation down regulates a serine protein kinase (Akt) involved in the regulation of the progression of cancer cells. The work suggests that the new developed nanocarrier for rapamycin could be used as radiosensitizer and could be used for local–regional or peripherical administration for the treatment of glioblastomas.

In the work entitled “*In silico* and *in vitro* evaluation of mimetic peptides as potential antigen candidates for prophylaxis of leishmaniasis,” Guedes et al. performed the liposomal encapsulation of synthetic peptides with antigenic activity against *Leishmania* spp. Six peptides obtained from *Leishmania* spp. membranes and histones were synthetized by the phage display technique. The entrapped peptide was administered to New Zealand rabbits and used to determine humoral immunogenic protection against *L. braziliensis* or *L. infantum*. The peptide mixture triggered IFN-γ, IL-12, IL-4, and TGF-β, which induced Th1 and Th2 cellular immune response by polarization of T CD4^+^ cells and high expression of iNOS in infected rabbits. These promissory results will be extended for the treatment of other pathologies and imply a new approach for the development of novel type of vaccines.

